# Simulating the Effect of Removing Circulating Tumor Cells (CTCs) from Blood Reveals That Only Implantable Devices Can Significantly Reduce Metastatic Burden of Patients

**DOI:** 10.3390/cancers16173078

**Published:** 2024-09-04

**Authors:** Werner Baumgartner, Nicola Aceto, Sebastian Lifka

**Affiliations:** 1 Institute of Biomedical Mechatronics, Johannes Kepler University of Linz, 4040 Linz, Austria; werner.baumgartner@jku.at; 2 Department of Biology, Institute of Molecular Health Sciences, ETH Zürich, 8093 Zurich, Switzerland; nicola.aceto@biol.ethz.ch

**Keywords:** circulating tumor cells (CTC), CTC cluster, CTC release dynamics, metastases, extracorporeal/intracorporeal blood purification devices, theoretical biological model

## Abstract

**Simple Summary:**

Circulating tumor cells (CTCs) play an important role in the formation of metastases in the body. Various approaches have been proposed to cleanse the blood of these CTCs and thus prevent the formation of metastases. However, the efficiency of non-implantable blood purification devices for CTC removal has not yet been clearly demonstrated. Here, we have attempted to evaluate, with a simple computer simulation, whether the use of a non-implantable device for CTC excision can be effective and lead to a significant reduction in metastases. We show that non-implantable devices for CTC removal are de facto ineffective in terms of reducing metastases due to various factors. The results of this work argue that only implantable devices can successfully deplete metastasis-relevant CTCs and potentially reduce the metastatic burden of cancer patients.

**Abstract:**

Circulating tumor cells (CTCs) are cells that have separated from a solid cancerous lesion and entered the bloodstream. They play a crucial role in driving the metastatic spread to distant organs, which is the leading cause of cancer-related deaths. Various concepts for blood purification devices aiming to remove CTCs from the blood and prevent metastases have been developed. Until now, it is not clear if such devices can indeed reduce new metastasis formation in a significant way. Here, we present a simple theoretical model of CTCs in the bloodstream that can be used to predict a reduction in metastatic burden using an extracorporeal or intracorporeal blood purification device. The model consists of a system of ordinary differential equations that was numerically solved and simulated. Various simulations with different parameter settings of extracorporeal and intracorporeal devices revealed that only devices implanted directly in tumor-draining vessels can reduce the metastatic burden significantly. Even if an extracorporeal device is used permanently, the reduction in metastases is only 82%, while a permanently operating implanted device in the tumor-draining vessel would achieve a reduction of 99.8%. These results are mainly due to the fact that only a small fraction of CTCs reaches peripheral circulation, resulting in a proportionally small amount of purified blood in extracorporeal devices.

## 1. Introduction

Cancer, a complex and heterogeneous disease, poses a formidable challenge to modern medicine. Central to its lethality is the process of metastasis, whereby cancer cells spread from the primary tumor to distant organs, seeding secondary tumors and ultimately compromising organ function. While much attention has historically been paid to the primary tumor, recent advances have highlighted the critical role of circulating tumor cells (CTCs) and their clusters in the metastatic cascade.

CTCs are cancer cells that have shed from the primary tumor into the bloodstream, representing a dynamic and heterogeneous population [1]. Unlike traditional biopsy methods, which sample a specific region of the tumor, CTC analysis offers a minimally invasive approach to monitoring disease progression and treatment response in real time. The presence of CTCs in peripheral blood serves as a biomarker of metastatic potential, with higher CTC counts correlating with an advanced disease stage and a poor prognosis [2]. The concept of CTCs was first proposed over a century ago by Australian physician Thomas Ashworth, who observed cancer cells circulating in the blood of patients with metastatic disease [3]. However, it wasn’t until recent decades, with the advent of sophisticated technologies and methodologies, that researchers could effectively isolate and characterize these elusive cells.

CTCs are not mere bystanders in the metastatic process; rather, they play a central role in driving metastatic spread, which has been conclusively shown in mouse models [4,5]. In patient samples, while of course CTC transplantation experiments from patient to patient cannot be performed, the abundance of CTCs has been prospectively correlated with both progression-free survival (PFS) and overall survival (OS) in several instances [2]. These cells possess remarkable adaptability, allowing them to survive in the hostile environment of the bloodstream, evade immune surveillance, and extravasate into distant tissues to establish secondary tumors. As such, CTCs represent attractive targets for therapeutic intervention and biomarker development.

Detecting and isolating CTCs from peripheral blood pose a significant technical challenge due to their rarity and heterogeneity. CTCs are typically present in minute quantities amidst a vast background of normal blood cells, necessitating highly sensitive and specific detection methods. Various approaches have been developed to overcome these challenges, leveraging advances in microfluidics, immunomagnetic separation, and molecular profiling techniques. One common strategy involves the use of epithelial cell adhesion molecule (EpCAM) antibodies to selectively capture CTCs expressing epithelial markers, as many solid tumors originate from epithelial tissues. However, this method may overlook mesenchymal-like phenotypes of CTCs, prompting the exploration of alternative markers and strategies for enrichment.

While individual CTCs have long been recognized as key players in metastasis, recent studies have shed light on the significance of CTC clusters—aggregates of two or more single CTCs found circulating in the bloodstream. These clusters exhibit distinct biological properties and enhanced metastatic potential compared to single CTCs, making them particularly intriguing targets for further investigation.

Once formed, CTC clusters exhibit increased resistance to anoikis (programmed cell death induced by detachment from the extracellular matrix) and immune surveillance, as well as enhanced adhesion to endothelial cells and extravasation into distant tissues.

The detection and characterization of CTC clusters hold profound clinical implications for cancer diagnosis, prognosis, and treatment. Like individual CTCs, the presence of CTC clusters in peripheral blood correlates with an advanced disease stage and a poor prognosis, underscoring their potential as prognostic biomarkers.

Furthermore, CTC clusters may serve as harbingers of imminent metastasis, providing valuable insight into the metastatic cascade and guiding therapeutic decision-making. Their enhanced metastatic potential and resistance to conventional therapies make them attractive targets for novel therapeutic strategies aimed at disrupting the metastatic process.

As research in the field of single CTCs and CTC clusters continues to evolve, several avenues of investigation hold promise for advancing our understanding and clinical management of cancer. Future endeavors aim to refine existing detection methods to enhance the sensitivity and specificity of CTC detection, particularly for rare and aggressive tumor subtypes.

Moreover, elucidating the molecular and functional characteristics of CTC clusters presents a compelling opportunity to identify novel therapeutic targets and develop innovative treatment modalities. By targeting CTCs and their clusters, researchers hope to disrupt the metastatic cascade and improve patient outcomes in advanced cancer.

In conclusion, single CTCs and CTC clusters represent dynamic and heterogeneous populations with profound implications for cancer biology and clinical practice. As our understanding of these cells deepens and technology continues to advance, single CTCs and their clusters hold the potential to revolutionize cancer diagnosis, prognosis, and treatment, ushering in a new era of precision oncology.

As metastasis is considered the main challenge in cancer, it is tempting to think of methods to remove single CTCs and CTC clusters from blood to reduce the metastatic burden [6]. There have been various ideas and research efforts focused on developing extracorporeal devices to remove circulating tumor cells from the blood as a means of preventing metastases. These approaches aim to capture and eliminate CTCs from circulation before they have the chance to establish secondary tumors in distant organs. Some of the strategies and concepts that have been explored in this area are microfluidic devices [7,8], immunomagnetic separation [9,10], filtration systems [11,12], dielectrophoresis [13,14], nanotechnologies [15,16], and biological interactions [17,18]. Even if the basic idea behind the majority of the work in this area is more for diagnostic purposes and identifying biomarkers [11,19,20,21], some researchers are considering using such devices for therapeutic purposes such as removing CTCs from the blood [8,22,23].

**Figure 1:**
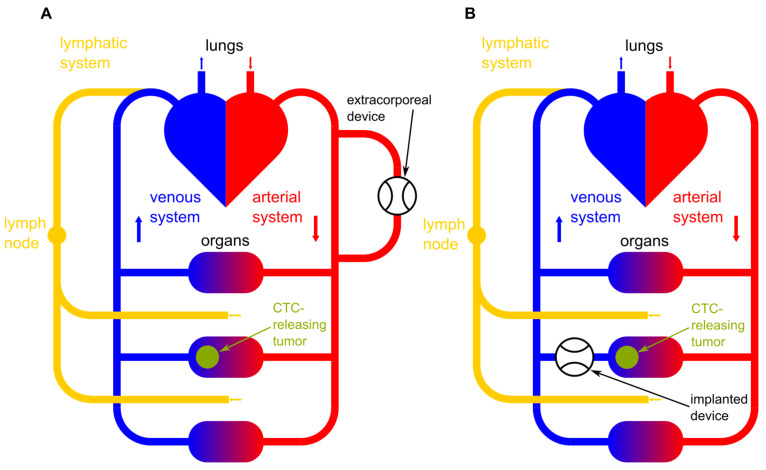
Schematic illustration of (**A**) an extracorporeal blood-clearing device clearing peripheral blood and (**B**) an implanted blood-clearing device right after the CTC-releasing tumor. Additionally, the lymphatic system as a possible bypass for CTCs around the device is pictured.

In addition to extracorporeal approaches, there is ongoing research into in vivo capture devices that can selectively target and remove CTCs directly from the bloodstream within the body [21]. These devices may be implanted or administered systemically to achieve localized CTC capture and removal. In Figure 1, a schematic illustration of an implanted device and an extracorporeal device are shown. Figure 1A shows an extracorporeal device clearing peripheral blood and Figure 1B shows an implanted device right after the CTC-releasing tumor. Additionally, the lymphatic system as one possible bypass for CTCs around the device is pictured.

Furthermore, some researchers are exploring combined therapeutic strategies that integrate CTC removal with other treatment modalities, such as chemotherapy or immunotherapy. By targeting CTCs in conjunction with primary tumor treatment, these approaches aim to prevent metastatic spread more effectively.

Significant progress has been made in developing CTC removal technologies and the concept of extracorporeal CTC removal holds promise as a potential strategy for preventing metastases and improving outcomes for cancer patients. However, it is not clear if extracorporeal devices or implantable devices can indeed reduce the metastatic potential significantly. For that purpose, we present here a simple theoretical model including new findings and parameter estimations that can predict the efficiency of extracorporeal and intracorporal devices removing certain percentages of single CTCs as well as CTC clusters from the bloodstream. The open-source program performing the simulation might predict if an approach has the potential to be of medical use and if so, it can help to optimize the design and parameters of operation.

## 2. Materials and Methods

### 2.1. Theoretical Modeling

For the theoretical description of the dynamic changes of single CTCs and CTC clusters in blood with and without a device selectively removing these events, a dynamic model has to be derived. To describe the system with a simple one-compartment model, we have to take care of the three system variables:

*n*(*t*) … total number of single CTCs in the blood compartment;

*c*(*t*) … total number of CTC clusters in the blood compartment;

*m*(*t*) … number of metastases.

The blood compartment is assumed to have a total volume *V_t_* and we assume a heart minute volume of *V_m_*. Thus, the concentrations of single CTCs and clusters can be determined to be *n*(*t*)/*V_t_* and *c*(*t*)/*V_t_*, respectively. For simplicity, we will assume clusters to have an average size (i.e., a constant average number of single CTCs aggregated). A more detailed analysis taking a distribution of sizes and polymerization–depolymerization dynamics into account appears not useful as the necessary parameters for the dynamics are experimentally not (yet) accessible and Aceto et al. [24] were able to show that circulating tumor cell clusters originate from oligoclonal tumor cell clusters and cannot be attributed to intravascular aggregation. Nevertheless, we will allow a simple aggregation in our model, which will be set to zero for most of our simulations. We now aim for a system of (delayed) ordinary differential equations:

(1) $$\begin{array}{c} \frac{\partial n}{\partial t}=r\left(t,m\left(t-\Delta t\right)\right)\cdot \left[\left(1-p_{lymph}\right)+p_{lymph}\cdot l_{pass}\right]+f_{dyn}\left(t,n\left(t\right),c\left(t\right)\right)+f_{dev}\left(t,n\left(t\right),c\left(t\right)\right),\\ \frac{\partial c}{\partial t}=p\left(t,m\left(t-\Delta t\right)\right)\cdot \left[\left(1-p_{lymphc}\right)+p_{lymphc}\cdot l_{passc}\right]+g_{dyn}\left(t,c\left(t\right)\right)+g_{dev}\left(t,c\left(t\right)\right),\\ \frac{\partial m}{\partial t}=q\left(t,n\left(t\right),c\left(t\right)\right)+r\left(t,m\left(t-\Delta t\right)\right)\cdot p_{lymph}\cdot l_{meta}+p\left(t,m\left(t-\Delta t\right)\right)\cdot p_{lymphc}\cdot l_{metac}. \end{array}$$

Here, *r* and *p* describe the release of single CTCs and clusters, respectively, from the primary tumor and from metastases. The latter is time-delayed as, hypothetically speaking, a newly established secondary tumor will not release CTCs. This delay time also includes the dormancy of the tumor cells, even if only with a single time constant. At this point, one could extend the model to a multi-state model and add additional cell populations. However, this would require detailed knowledge of the kinetic scheme and additional parameters, which are currently unavailable. Therefore, for the sake of simplicity, this was not considered in more detail; a simple delay time is sufficient, as will be shown below.

The functions *f_dyn_* and *g_dyn_* describe the dynamic changes due to natural cell death, aggregation, blood shear stress, etc., of single and clustered CTCs, respectively. The functions *f_dev_* and *g_dev_* describe the changes due to a device capable of removing single CTCs and/or clusters from blood.

In addition, a bypass for the CTCs via the lymphatic system is to be included in the model. CTCs that enter the lymphatic system from the bloodstream cannot be destroyed by the device, but can certainly form metastases in the lymph nodes, for example, and also return to the bloodstream. The parameters *p_lymph_* and *p_lymphc_* represent the ratio (between 0 and 1) of single and clustered CTCs which are released into the lymphatic system; *l_pass_* and *l_passc_* denote the probabilities of single and clustered CTCs that pass from the lymphatic system into the blood. The two parameters *l_meta_* and *l_metac_* describe the probability of CTCs forming metastases in the lymphatic system and are calculated as $l_{meta}=1-\left(l_{pass}+l_{dead}\right)$ and $l_{metac}=1-\left(l_{passc}+l_{deadc}\right)$, respectively, whereby *l_dead_* and *l_deadc_* represent the probability of CTCs being destroyed in the lymphatic system.

The release *r*(*t, m*(*t* − Δ*t*)) and *p*(*t, m*(*t* − Δ*t*)) can take place at the primary tumor and, with a certain time delay Δ*t*, from the metastases. As a first and simple approach, one could consider a constant release of events (single CTCs and CTC clusters) over time. However, Diamantopoulou et al. [25] have shown that the release of events in human and mice changes with a circadian rhythm significantly. In our model we assumed

(2) $$\begin{array}{c} r\left(t,m\left(t-\Delta t\right)\right)=\left\{r_{const}+\frac{r_{dyn}}{2}\cdot \left[1+\cos\left(2\cdot \pi \cdot \frac{t+T_{dev}}{T_{day}}\right)\right]\right\}\cdot \left[1+M\cdot m\left(t-\Delta t\right)\right],\\ p\left(t,m\left(t-\Delta t\right)\right)=\left\{p_{const}+\frac{p_{dyn}}{2}\cdot \left[1+\cos\left(2\cdot \pi \cdot \frac{t+T_{dev}}{T_{day}}\right)\right]\right\}\cdot \left[1+M\cdot m\left(t-\Delta t\right)\right], \end{array}$$

where *r_const_* ≥ *r_dyn_* and *p_const_* ≥ *p_dyn_*. Thus, we have a constant release of single and clustered CTCs onto which a cosine function with period of one day is modulated. The cosine shape was chosen since it is a simple way of modeling a wave-like cardiac rhythm. For practical purposes for the simulation, we assumed that the device removing events from blood started its work at *t* = 0. Thus, the release was shifted by a time *T_dev_*. *M* is a weighting factor representing the ratio of primary tumors to metastases and *T_day_* represents the duration of a day, in our case expressed in minutes.

This simple release model does not take tumor growth, and thus increasing releases over a long time, into account, as this is not of importance to the question of whether or not technical devices can reduce metastatic burden in patients. However, the model could be simply improved for other applications by replacing the 1 in the round brackets with a time-dependent function describing the growth of the primary tumor and the *M* by a *M*(*t*) describing the growth of metastases.

Aceto et al. [24] were able to show that circulating tumor cell clusters originate from oligoclonal tumor cell clusters and cannot be attributed to the intravascular aggregation of single CTCs. Thus, there is no growth term involving *n²* or *nc* in the differential equations. However, if tumors are found with associated CTCs that have an increased tendency to polymerize, an additional source of *c*(*t*) can be simply included. For simplicity, this possibility was neglected.

Due to natural cell death, especially due to the mechanical stress in the blood system, the numbers of single CTCs, as well as clusters, are changing. This is accounted for in the functions *f_dyn_*(*n*(*t,c*(*t*),*t*)) and *g_dyn_*(*n*(*t,c*(*t*),*t*)), respectively:

(3) $$\begin{array}{c} f_{dyn}\left(t,n\left(t\right),c\left(t\right)\right)=-n\left(t\right)\cdot \left(k_{met}+k_{dead}\right)+c\left(t\right)\cdot k_{deadc}\cdot N_{c},\\ g_{dyn}\left(t,c\left(t\right)\right)=-c\left(t\right)\cdot \left(k_{metc}+k_{deadc}\right). \end{array}$$

Here, *k_met_* denotes the rate at which single CTCs extravasate and form metastases and *k_dead_* denotes the rate of single CTCs dying per unit of time due to natural reasons. Similarly, *k_metc_* and *k_deadc_* are the equivalent rates for clustered CTCs. If a cluster is disrupted due to hemodynamic shear stress, the cells in the cluster either die or are set free as single CTCs. To take this into account, the parameter *N_c_* is introduced. This is the number of cells (single CTCs) that are set free and survive on average if a cluster is disrupted. Thus, if *N_c_* > 0 the clusters that are disrupted are a source for new single CTCs. From this, we can conclude the formation of metastases to be

(4) $$q\left(t,n\left(t\right),c\left(t\right)\right)=k_{met}\cdot n\left(t\right)+k_{metc}\cdot c\left(t\right).$$

Finally, if a device is introduced that removes single CTCs and CTC clusters from blood, we have to distinguish two cases:

1. The device acts like a dialysis machine and treats peripheral blood;
2. The device is implanted and removes CTCs from the venous blood right after the organ exhibiting the primary tumor.

In both cases, we obtain

(5) $$\begin{array}{c} f_{dev}\left(t,n\left(t\right),c\left(t\right)\right)=R_{vol}\cdot \left[-n\left(t\right)\cdot eff_{dev}+c\left(t\right)\cdot eff_{devc}\cdot N_{cdev}\right],\\ g_{dev}\left(t,c\left(t\right)\right)=-R_{vol}\cdot c\left(t\right)\cdot eff_{devc}. \end{array}$$

Here, *R_vol_* is the volume percentage of the heart minute volume that passes the device in a minute. *eff_dev_* is the efficiency of the device (i.e., the ratio of CTCs being killed by the device to the CTCs entering the device). Similarly, for clusters, we define the efficiency of the device to clear clusters as *eff_devc_*. Also, here we allow a certain average number *N_cdev_* of cells to survive and to be set free.

If the device is implanted and handles the venous blood after the primary tumor, the above equations are still valid, but we have to modify the release Equation (2) as

(6) $$\begin{array}{c} r\left(t,m\left(t-\Delta t\right)\right)=\left\{r_{const}+\frac{r_{dyn}}{2}\cdot \left[1+\cos\left(2\cdot \pi \cdot \frac{t+T_{dev}}{T_{day}}\right)\right]\right\}\cdot \left[1-eff_{dev}+M\cdot m\left(t-\Delta t\right)\right],\\ p\left(t,m\left(t-\Delta t\right)\right)=\left\{p_{const}+\frac{p_{dyn}}{2}\cdot \left[1+\cos\left(2\cdot \pi \cdot \frac{t+T_{dev}}{T_{day}}\right)\right]\right\}\cdot \left[1-eff_{devc}+M\cdot m\left(t-\Delta t\right)\right]. \end{array}$$

This is due to the fact that the CTCs released from the tumor must pass the device before they are released into the body, and therefore are immediately reduced by the device. This is not the case in extracorporeal devices.

### 2.2. Simulation Method

The above derived system of ordinary differential equations (ODEs) is, in the present form, a system of linear delayed ODEs. If we neglect the release due to metastases, we end up with an inhomogeneous system of linear ODEs. This could be solved analytically, in principle. However, as the inhomogeneity of the release and a non-steady inhomogeneity of the device (that can be switched on and off periodically) result in cumbersome integrations, a numerical solution of the system of ODEs was established. An improved Euler algorithm was implemented in Python v3 code that can be found in a Zenodo repository [26] and visualized with matplotlib [27]. The explicit Euler method (also referred to as the Euler–Cauchy method or Euler-forward method) is the simplest method for the numerical solution of an initial value problem introduced by Leonhard Euler in 1768 in his book *Institutiones Calculi Integralis* [28]. For the numerical solution of the initial value problem:

(7) $$y\prime =f\left(t,y\right),y\left(t_{0}\right)=y_{0},$$

or of ODEs choose a discretization step size *h* > 0, consider the discrete time points $t_{k}=t_{0}+k\cdot h,k=0,\;1,\;2,\;\ldots$ and calculate the values

(8) $$y_{k+1}=y_{k}+h\cdot f\left(t_{k},y_{k}\right).$$

The calculated values *y_k_* represent approximations of the actual values *y*(*t_k_*) of the exact solution of the initial value problem.

To obtain even more precise results for a given step size *h*, the so-called improved Euler method or midpoint method can be employed. Here, the slope (derivative) of the function for the interval *t_k_* to *t_k+1_* is not simply *f*(*t_k_*), but *f*(*t_k_ + h/2*). Thus, the iterative calculations for the *y*-values are defined as

(9) $$y_{k+1}=y_{k}+h\cdot f\left(t_{k}+\frac{h}{2},y_{k+1/2}\right)\mathrm{with}\;y_{k+1/2}=y_{k}+\frac{h}{2}\cdot f\left(t_{k},y_{k}\right).$$

The local error at each step of the midpoint method is of order O(h^3^). Thus, while more computationally intensive than the simple Euler method, the midpoint method’s error generally decreases faster as *h* tends toward 0.

### 2.3. Determination/Estimation of the Model Parameters

#### 2.3.1. CTC Death Rate *k_dead_*

This rate can be estimated, for example, from experiments by Meng et al. [29], who measured the CTC concentration in blood after tumor removal in different human patients. Half-life *τ*_0.5_ was found to be in the range of 1–2.5 h. From the relation

(10) $$k_{dead}=\frac{\ln\left(2\right)}{\tau_{0.5}},$$

we can obtain a range for *k_dead_* to be approximately 0.01 to 0.004 min^−1^.

#### 2.3.2. CTC Release Rates *r_const_* and *r_dyn_*

Measurements of CTC numbers in samples of peripheral blood taken over a day indicate that typically [CTC] = 0.1–5 CTCs per ml blood, dependent on the type of cancer and the stage [25,29,30]. As throughout the day, the release was shown to be often minimal [25], the release rate *r_const_* can be estimated from the equilibrium assumption

(11) $$\frac{dn}{dt}=0=r_{const}-n\cdot k_{dead}=r_{const}-\left[CTC\right]\cdot V_{tot}\cdot k_{dead}\;\to \;r_{const}\approx \left[CTC\right]\cdot V_{tot}\cdot k_{dead},$$

with *V_tot_* being the total blood volume. This volume is approximately 5 L for an adult male patient.

As also shown by Diamantopoulou et al. [25], the circadian change of measurable numbers of CTCs ranges from a factor of 1 to 100 times (i.e., the amplitude of changes is 1–100 times the minimal number of CTCs throughout a day). As the minimal number corresponds to *r_const_*, *r_dyn_* can be estimated to be 1–100 · *r_const_*, accordingly.

#### 2.3.3. Clusters’ Dead and Release Rates *k_deadc_*, *p_const_*, and *p_dyn_*

Clusters appear to be released at similar or even higher rates, but their circulation half-life is much lower [24]. In order to fit the observations in [24], a half-life of 1 to 30 min can be assumed from which the rate *k_deadc_* can be determined.

#### 2.3.4. Parameters for Devices Clearing Blood *T_dev_*, Δ*T_dev_*, *R_vol_*, *eff_dev_* and *eff_devc_*

The time during which the device is on during day *T_dev_* can be set freely from 0 to 24 h. The time delay Δ*T_dev_* describes the time difference of the device activation and the maximum of CTC release. The volume ratio of blood that can be cleared is the volume per minute that passes the device over the heart minute volume. Here, we assume a heart minute volume of 5 L/min (i.e., the total blood volume is circulated once a minute, which is reasonable during rest). If a device “cleans” peripheral venous blood, we can assume that 50–100 mL per minute can be treated, which corresponds to blood flow during blood donation. If, however, an arteriovenous shunt is surgically introduced, we can calculate using 500 mL/min, which corresponds to typical values for hemodialysis. For implantable devices, the ratio depends on the perfusion of the organ where the device is implanted. We assumed that the organ takes blood perfusion on the same order of magnitude as a peripheral device. The efficiencies of single- and clustered-CTC removal *eff_dev_* and *eff_devc_* strongly depend on the device. Typically, we assumed that 90–100% of the single CTCs will be killed, corresponding to *eff_dev_* being 0.9 to 1, and we further assumed that 99 to 100% of clusters would be destroyed, corresponding to *eff_devc_* being 0.99 to 1.

#### 2.3.5. Single CTCs Set Free from Destroyed Clusters *N_c_* and *N_cdev_*

If a CTC cluster is destroyed, either the cells are killed or are, in part, set free as single CTCs. To take this into account, *N_c_* and *N_cdev_* were introduced, the number of single CTCs set free from one single destroyed cluster. Here, *N_c_* is the number of single CTCs set free from a cluster destroyed physiologically by normal circulation, and *N_cdev_* is the number of single CTCs set free from a cluster destroyed by the device. Based on observations from the previous work [22], these parameters were set to be equal to anywhere from 0 to 15 in our simulations.

#### 2.3.6. Rate Constant of Metastases Formation *k_met_* and *k_metc_*

These rate constants are almost impossible to determine as there are no reliable quantitative literature data available. We only know from [24] that the metastatic potential of clusters is about 50 times higher than that of single CTCs. As only a minimal fraction of single CTCs actually forms clusters [31], the decrease in single and clustered CTCs due to metastases formation can be neglected. Even though absolute values would be of interest, a qualitative statement about the metastatic burden in a patient is also valuable. Equation (6) for the metastases is the dead end and is linearly dependent on the rate constants. Thus, the principal behavior will be the same for each small value of *k_met_* and *k_metc_*, as long as the reduction in *n*(*t*) and *c*(*t*) is negligible. The absolute numbers differ by a constant multiplicative factor. For that reason, we decided to set *k_met_* = 10^−8^ and *met_c_* to be 50 times *k_metc_*.

#### 2.3.7. Time Delay for Metastases to Emit CTCs Δ*t*

This value is also hard to estimate. For our simulations, we assumed 20 days.

#### 2.3.8. Parameters for the Lymphatic System *p_lymph_*, *p_lymphc_*, *l_dead_*, *l_deadc_*, *l_pass_*, *l_passc_*, *l_meta_*, and *l_metac_*

The parameters for the lymphatic system are difficult to quantify; in this model, they are currently most likely to be used to run through various scenarios of a bypass for CTCs via the lymphatic system. *p_lymph_* and *p_lymphc_* are the ratios of single CTCs and clustered CTCs, respectively, released into the lymphatic system. They range between 0 and 1; 0 means no release into the lymphatic system and 1 means all CTCs are released into the lymphatic system. The exact values are hardly accessible and may depend on the kind of tumor itself. We assumed these two parameters to range between 0 and 0.3, as we thought these could be realistic values. The parameters *l_dead_* and *l_deadc_* are the probabilities of single CTCs and clustered CTCs to be destroyed in the lymphatic system. Again, these parameters are not available in the literature. As a starting point, we decided to set them to a rather high value, more specifically *l_dead_* = 0.99 and *l_deadc_* = 0.9999. *l_pass_* and *l_passc_* denote the probabilities of CTCs passing from the lymphatic system back into the bloodstream. As a starting point, these parameters were chosen to be rather low. *l_pass_* was chosen to be 0.0095 and *l_passc_* was set to zero, as we assumed that no clustered CTCs can enter the bloodstream from the lymphatic system. Finally, *l_meta_* and *l_metac_* represent the probability of single and clustered CTCs to form metastases in the lymphatic system. The values for these two parameters result from the probabilities of CTCs to be destroyed or to pass from the lymphatic system into the bloodstream. They are calculated as $l_{meta}=1-\left(l_{pass}+l_{dead}\right)$ and $l_{metac}=1-\left(l_{passc}+l_{deadc}\right)$, respectively.

## 3. Results

**Figure 2:**
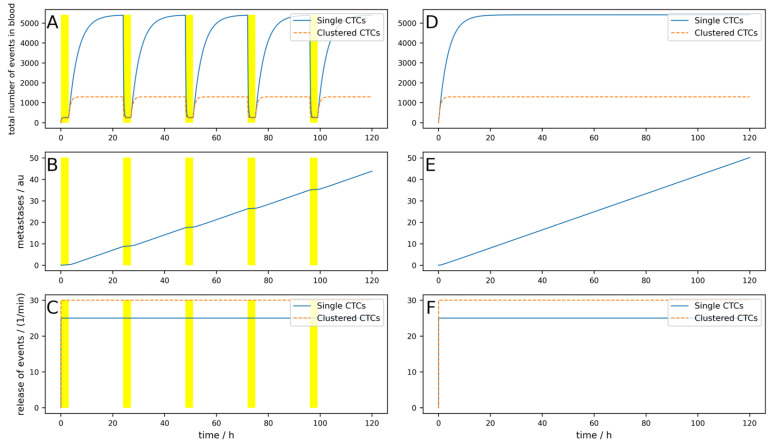
Simulation of a constant release of single and clustered CTCs over 5 days. (**A**–**C**) A device that clears peripheral blood daily for 3 h, indicated by the yellow areas, is simulated. (**D**–**F**) Control without clearing device using the same parameters. (**A**,**D**) Dynamic behavior of single and clustered CTCs in absolute numbers. (**B**,**E**) Metastatic burden in arbitrary units. (**C**,**F**) Release of single and clustered CTCs from the primary tumor.

To first check if the behavior of the model is as expected, we simulated a constant release of *r_const_* = 25 min^−1^ and *r_dyn_* = 0 min^−1^, as well as *p_const_* = 30 min^−1^ and *p_dyn_* = 0 min^−1^. CTCs were assumed to have a rather high half-life of 150 min, corresponding to *k_dead_* = 0.0046, and the clusters were assumed to have a half-life of 30 min, corresponding to *k_deadc_* = 0.023. This corresponds to a rather slow dynamic which would be in favor of a CTC-clearing device, as will be shown below. We neglected CTCs due to release from secondary tumors (i.e., *M* = 0). A patient with 5 L total blood volume was assumed to have an arteriovenous shunt; thus, 0.5 L/min blood can be cleared. We simulated 5 days, and each day, a device was applied for 3 h that could clear 90% of the single CTCs and 99% of clusters. Thus, we obtained *T_dev_* = 3 h, *R_vol_* = 0.1, *eff_dev_* = 0.9, and *eff_devc_* = 0.99. *N_c_* and *N_cdev_* were set to 0. The bypass over the lymphatic system was neglected for the first couple of simulations and is only taken into account toward the end of Section 3. The results are shown in Figure 2. Figure 2A–C shows the simulation with a blood-clearing device; Figure 2D,F shows a control simulation without a device using the same parameters. The yellow highlighted areas on the left indicate the switch-on time of the device, in other words, *T_dev_*. The most interesting aspect is the comparison of the metastatic burden at the end of the simulation period, so to say, the comparison between Figure 2B,E. One can see that due to the clearing device, the metastatic burden in Figure 2B at the end of the simulation time is slightly lower than in Figure 2E without the clearing device. The percentage reduction in the metastatic burden compared to the control can then be calculated from the absolute values of the metastases in arbitrary units at the end of the respective simulation time, whereby the absolute value itself has no relevance. All the following figures are structured identically; only a few parameters are changed to play through different scenarios.

**Figure A1:**
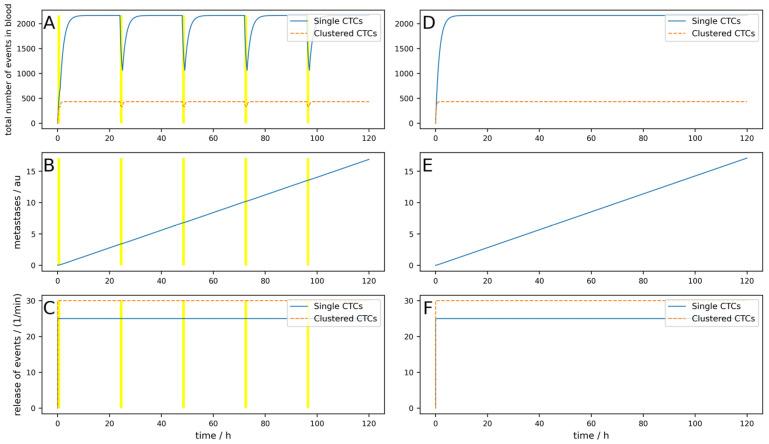
Simulation of a constant release of single and clustered CTCs over 5 days. (**A**–**C**) A device that clears peripheral blood daily for 1 h, indicated by the yellow areas, is simulated. (**D**–**F**) Control without clearing device using the same parameters. (**A**,**D**) Dynamic behavior of single and clustered CTCs in absolute numbers. (**B**,**E**) Metastatic burden in arbitrary units. (**C**,**F**) Release of single and clustered CTCs from the primary tumor.

The metastases only decrease by 12%, and even though an arteriovenous shunt was placed, the CTCs have very slow dynamics (long half-life) and the device is used for 3 h each day. If, for example, the cells have a half-life of 1 h and the clusters a half-life of 10 min, only 100 mL/min blood can be cleared and if the device is applied for an hour daily, the reduction would be only 1%. This simulation is shown in Figure A1 in Appendix A.

**Figure A2:**
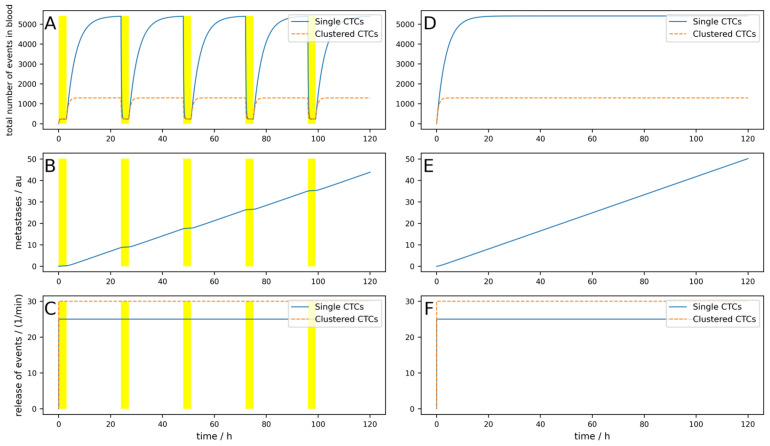
Simulation of a constant release of single and clustered CTCs over 5 days. (**A**–**C**) A device that clears peripheral blood daily for 3 h with 100% efficiency, indicated by the yellow areas, is simulated. (**D**–**F**) Control without clearing device using the same parameters. (**A**,**D**) Dynamic behavior of single and clustered CTCs in absolute numbers. (**B**,**E**) Metastatic burden in arbitrary units. (**C**,**F**) Release of single and clustered CTCs from the primary tumor.

Even if the device worked perfectly (i.e., 100% of single CTCs and CTC clusters are removed from the blood) and the circumstances were excellent, as in the simulation shown in Figure 2, the reduction in metastases is only 12.7% (Figure A2).

**Figure 3:**
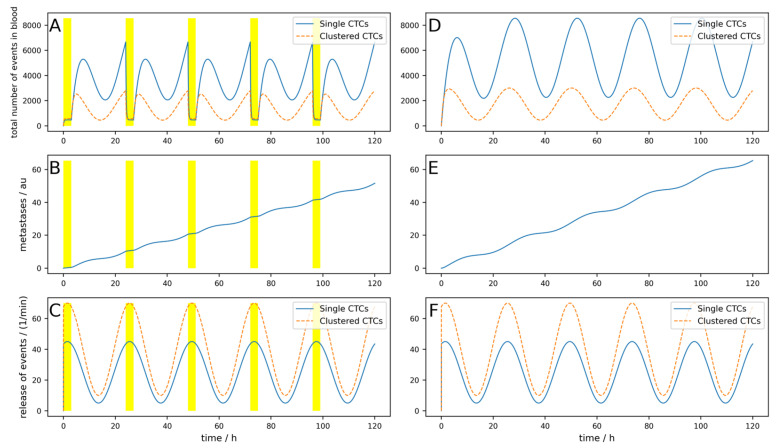
Simulation of an oscillating release of single and clustered CTCs over 5 days. (**A**–**C**) A device that clears peripheral blood daily for 3 h, right at the maximal release, indicated by the yellow areas, is simulated. (**D**–**F**) Control without clearing device using the same parameters. (**A**,**D**) Behavior of single and clustered CTCs in absolute number. (**B**,**E**) Metastatic burden in arbitrary units. (**C**,**F**) Release of single and clustered CTCs from the primary tumor.

The release of CTCs is, at least for some tumors, not constant over the day. In Figure 3, a simulation of 5 days is shown, where a base release of five single CTCs per minute and a peak-to-peak amplitude of 40 single CTCs per minute, as well as base release of 10 clusters per minute and a peak-to-peak amplitude of 60 clusters per minute, are simulated. If these parameters are chosen like this, the simulations fit the observations made in [25]. The device is switched on symmetrically around the maximum of the release (i.e., Δ*T_dev_* = 0) for 3 h. Of course, as the peak occurs during nighttime, this is a quite uncommon time for a clinical treatment. However, this assumption is not so far-fetched when considering that there are also night dialysis treatments. All other parameters are the same as for the simulation in Figure 3.

The conditions for a device are assumed to be nearly ideal (i.e., the device is used right at the maximal release and the cell dynamics are rather low). Nevertheless, the reduction in the metastatic burden is only 21%.

**Figure A3:**
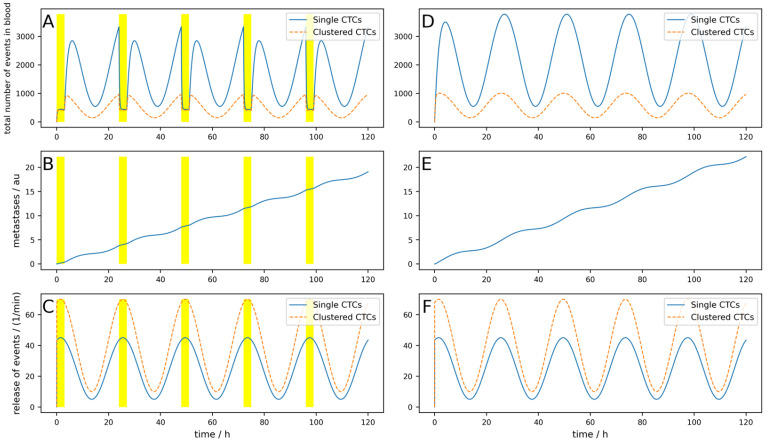
Simulation of a high dynamic oscillating release of single and clustered CTCs over 5 days. (**A**–**C**) A device that clears peripheral blood daily for 3 h, right at the maximal release, indicated by the yellow areas, is simulated. (**D**–**F**) Control without clearing device using the same parameters. (**A**,**D**) Behavior of single and clustered CTCs in absolute numbers. (**B**,**E**) Metastatic burden in arbitrary units. (**C**,**F**) Release of single and clustered CTCs from the primary tumor.

**Figure A4:**
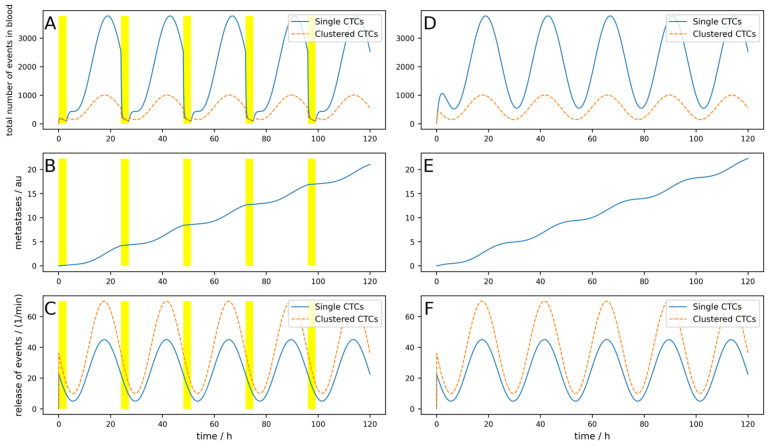
Simulation of a high dynamic oscillating release of single and clustered CTCs over 5 days. (**A**–**C**) A device that clears peripheral blood daily for 3 h, 8 h after the maximal release, indicated by the yellow areas, is simulated. (**D**–**F**) Control without clearing device using the same parameters. (**A**,**D**) Behavior of single and clustered CTCs in absolute numbers. (**B**,**E**) Metastatic burden in arbitrary units. (**C**,**F**) Release of single and clustered CTCs from the primary tumor.

If the half-life of the cells were shorter, corresponding to higher dynamics as simulated in Figure A3, the result is even worse, and metastases are only reduced by 14%. Furthermore, if the device is not applied during the maximal release (which is normally in the middle of the night), but 8 h later as simulated in Figure A4, only a 5.5% reduction can be reached, and the benefits are therefore very limited.

**Figure A5:**
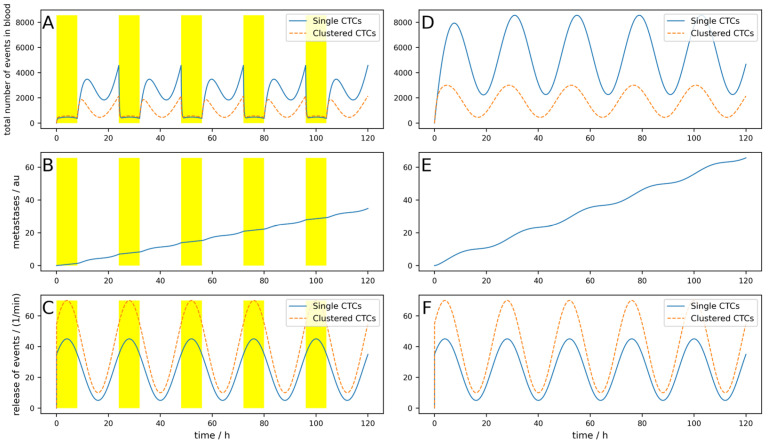
Simulation of an oscillating release of single and clustered CTCs over 5 days. (**A**–**C**) A device that clears peripheral blood daily for 8 h (e.g., overnight), right at the maximal release, indicated by the yellow areas, is simulated. (**D**–**F**) Control without clearing device using the same parameters. (**A**,**D**) Behavior of single and clustered CTCs in absolute numbers. (**B**,**E**) Metastatic burden in arbitrary units. (**C**,**F**) Release of single and clustered CTCs from the primary tumor.

If the device is used all night, e.g., for 8 h a day when the tumor exhibits low dynamics (like in Figure A3), the reduction reaches 47% (Figure A5).

**Figure A6:**
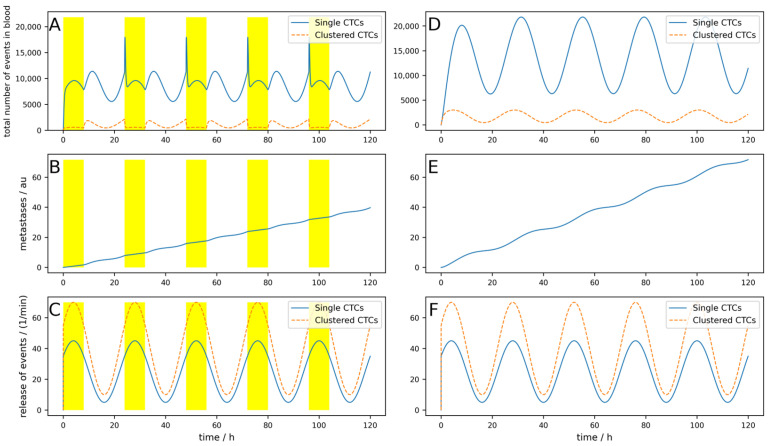
Simulation of an oscillating release of single and clustered CTCs over 5 days. (**A**–**C**) A device that clears peripheral blood daily for 8 h (e.g., overnight), right at the maximal release, indicated by the yellow areas and releases single CTCs, is simulated. (**D**–**F**) Control without clearing device using the same parameters. (**A**,**D**) Behavior of single and clustered CTCs in absolute numbers. (**B**,**E**) Metastatic burden in arbitrary units. (**C**,**F**) Release of single and clustered CTCs from the primary tumor.

Interestingly, if the device does not simply destroy the clusters and kill all cells but releases some single CTCs, a little peak in the number of CTCs can be observed at the onset of the device (Figure A6). The reduction in metastases is still similar, 45% in this case.

**Figure 4:**
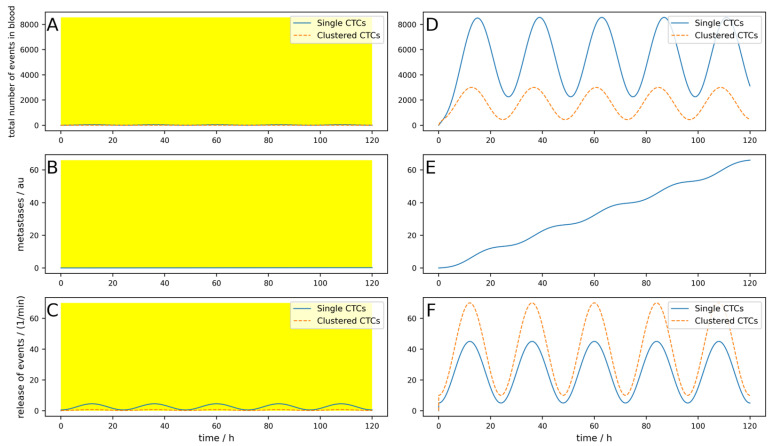
Effect of an implantable device clearing the venous blood at the tumor-draining vessel over 5 days. (**A**–**C**) A device that clears venous blood at the tumor-draining vessel is simulated. The device works 24 h a day (indicated by the yellow area). (**D**–**F**) Control without clearing device using the same parameters. (**A**,**D**) Behavior of single and clustered CTCs in absolute numbers. (**B**,**E**) Metastatic burden in arbitrary units. (**C**,**F**) Release of single and clustered CTCs from the primary tumor.

**Figure A7:**
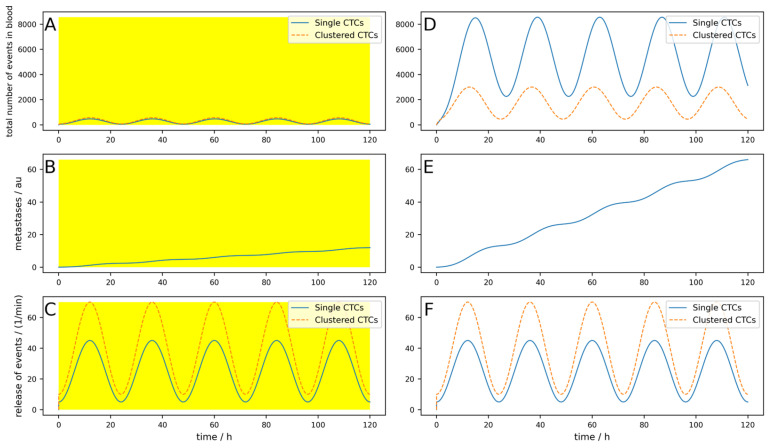
Simulation of an oscillating release of single and clustered CTCs over 5 days. (**A**–**C**) A device that clears peripheral blood all day long for 24 h, indicated by the yellow areas, is simulated. (**D**–**F**) Control without clearing device using the same parameters. (**A**,**D**) Behavior of single and clustered CTCs in absolute numbers. (**B**,**E**) Metastatic burden in arbitrary units. (**C**,**F**) Release of single and clustered CTCs from the primary tumor.

If the device is portable and a person can use it permanently (24/7), as simulated in Figure A7, a reduction of 82% can be reached. If a device can be implanted into a patient directly in the tumor-draining vessel, the effects are different, as shown in Figure 4. The parameters are equal to the Figure A7 simulation, except the location of the 24/7 running device. Here, a 99.8% reduction in the metastatic burden is reached.

**Figure A8:**
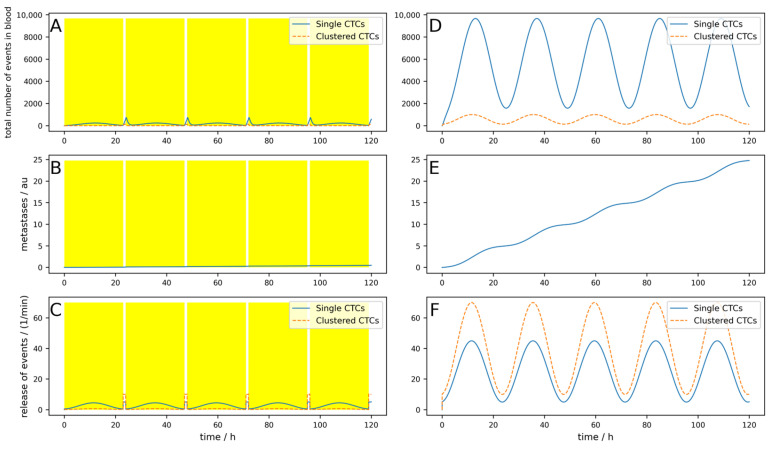
Simulation of a high dynamic oscillating release of single and clustered CTCs over 5 days. (**A**–**C**) An implantable device that clears venous blood at the tumor-draining vessel daily for 23 h (indicated by the yellow areas) and releases single CTCs, is simulated; (**D**–**F**) Control without clearing device using the same parameters. (**A**,**D**) Behavior of single and clustered CTCs in absolute numbers. (**B**,**E**) Metastatic burden in arbitrary units. (**C**,**F**) Release of single and clustered CTCs from the primary tumor.

Even if the implantable device is not working perfectly and the parameters are unfavorable (high dynamics, the device works only 23 h a day, single CTCs are set free upon cluster destruction, and only 100 mL per minute are cleared), a reduction of 98.1% is reached (Figure A8).

**Figure 5:**
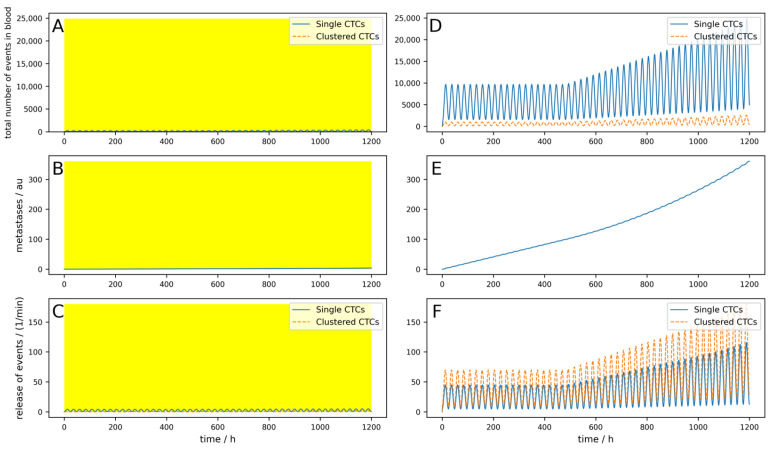
A 50-day simulation of an implantable device where the metastases can emit CTCs. (**A**–**C**) A device that clears venous blood at the tumor-draining vessel is simulated. The device works 24 h a day (indicated by the yellow area). (**D**–**F**) Control without clearing device using the same parameters. (**A**,**D**) Behavior of single and clustered CTCs in absolute numbers. (**B**,**E**) Metastatic burden in arbitrary units. (**C**,**F**) Release of single and clustered CTCs from the primary tumor and the metastases.

**Figure 6:**
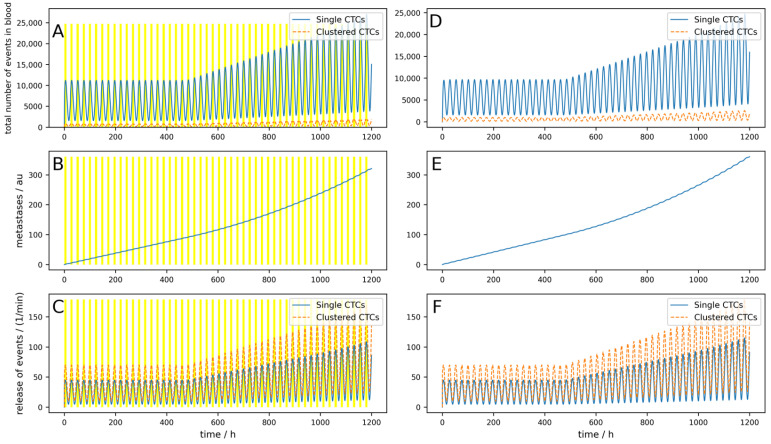
A 50-day simulation of an extracorporeal device where the metastases can emit CTCs. (**A**–**C**) A device that clears peripheral blood is simulated. The device works 8 h a day (indicated by the yellow areas). (**D**–**F**) Control without clearing device using the same parameters. (**A**,**D**) Behavior of single and clustered CTCs in absolute numbers. (**B**,**E**) Metastatic burden in arbitrary units. (**C**,**F**) Release of single and clustered CTCs from the primary tumor and the metastases.

Even for longer times under these suboptimal conditions and with the possibility that metastases can, after a time delay Δ*t* = 20 days emit CTCs, the implantable device shows good results (i.e., a reduction in metastases of 99%, as depicted in Figure 5). Under the same conditions, an 8 h a day working extracorporeal device only shows a reduction of 11%. This is depicted in Figure 6.

The parameters of all simulations shown in Figure 2, Figure 3, Figure 4, Figure 5 and Figure 6 and Figure A1, Figure A2, Figure A3, Figure A4, Figure A5, Figure A6, Figure A7 and Figure A8 are listed in Table 1. For clarity, Table A1 lists all the parameters of the simulations shown in Figure A1, Figure A2, Figure A3, Figure A4, Figure A5, Figure A6, Figure A7 and Figure A8. The underlying data can be found in the Zenodo repository [26].

**Figure 7:**
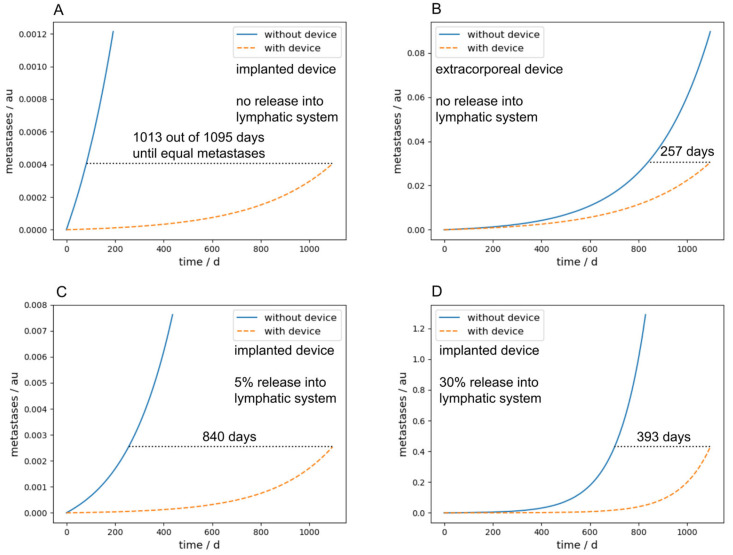
Comparison of the metastatic burden after a long-term simulation of three years between implanted and extracorporeal devices. The curves in blue show the progress of the metastases without the device and the orange dashed curve shows the course of metastases with the device. The number of days shown in each panel describes the time until the same number of metastases is reached with the clearing device in comparison to without the device. (**A**) Simulation of an implanted device without release into the lymphatic system. (**B**) Simulation of an extracorporeal device without release into the lymphatic system. (**C**) Simulation of an implanted device with a 5% release into the lymphatic system. (**D**) Simulation of an implanted device with a 30% release into the lymphatic system.

Figure 7A,B compare the metastatic burden between implanted and extracorporeal devices after a long-term simulation of three years, and therefore summarize the main findings of the paper. The parameters were chosen identically in (A) and (B) and are the same as used in the simulation in Figure 5, with exception of *N_c_* and *N_cdev_*, which were set to zero for the sake of simplicity and *M* was set to 0.001. The number of days in the diagram describes the time in which it takes longer with the device to achieve the same metastasis as without the device. One can see that with an intracorporeal device, this time is 1013 days, four times as long as with an extracorporeal device, which takes 257 days.

Furthermore, if a bypass, for example over the lymphatic system in our case, is considered, the performance of an implanted device is significantly decreased due to the fact that the CTCs transported over the lymphatic system cannot be cleared by the device. Figure 7C,D show this scenario with a release rate into the lymphatic system of 5% and 30%, respectively. With an implanted device and a release rate of 30% into the lymphatic system, the time until the same metastatic burden as without any device is reached is 393 days, two and a half times lower as without any release into the lymphatic system. The parameters for Figure 7C,D were chosen identically to Figure 7A,B; the lymphatic parameters were chosen as follows: *p_lymph_* = *p_lymphc_* = 0.05 or 0.3, respectively, *l_dead_* = 0.99, *l_deadc_* = 0.9999, *l_pass_* = 0.0095, and *l_passc_* = 0. A similar effect occurs in implanted devices if *M* gets higher. Since this means that metastases in turn emit CTCs, the implanted devices act more and more like an extracorporeal device.

## 4. Discussion

The release of single and clustered CTCs appears to be a key element in metastasis formation [1,4,5,24,31]. The popular idea of technical devices capable of removing CTCs from blood and thus reducing the metastatic burden is tempting and has been proposed several times in the past [8,11,21,22,23]. Most of these cited works were primarily aimed at other aspects, like enriching CTCs for diagnostic purposes but raised the idea of using devices to clean the blood from CTCs. The proposed devices, although different in their exact functionality, can be divided into two approaches: extracorporeal and implantable. The extracorporeal devices should act like a hemodialysis machine or plasmaphoresis, while the implantable should be placed right at the tumor-draining vessel. A quantitative estimation of the resulting effect (i.e., of the reduction in the metastatic burden) has been missing so far.

Various aspects of the process of release, distribution in the vascular system [32], destruction, adhesion, and extravasation [33] of single and clustered CTCs were the aim of theoretical modeling. Here, we aimed for a simple one-compartment model of single CTCs and CTC clusters in blood that takes the effects of such blood-clearing devices into account. One disadvantage of such a model is, of course, the fact that the distribution of CTCs throughout the body is assumed to be uniform, which is not necessarily the case in reality. Furthermore, the clearance is assumed to be linear, as no distribution parameter is used. Since CTCs are distributed throughout the body, one could argue that a multi-compartment model might be more appropriate; however, Meng et al. [29] were able to show that the behavior of the CTC concentration in blood can be reasonably well described with a one-compartment model. Of course, a much more sophisticated model could be developed. However, this would go hand-in-hand with more model parameters, such as tumor size, cellularity, vasculature (hypoxic nature), and the microenvironment, together with treatment status and many more. As these additional parameters are, in our opinion, not that important for the use case in this paper, they were neglected for sake of simplicity. Of course, for other use cases, these parameters may be of major importance.

As already described above, several parameters are not available yet from experiments directly. Some of the parameters needed for the model derived here, like the rate of CTC release, had to be determined indirectly. Some parameters had to be guessed, like the number of single CTCs that are set free from a destroyed cluster, and for some parameters, like the rate of metastasis formation, not even the order of magnitude is known. The latter is not a big problem, as the metastatic burden linearly depends on this rate and thus the relative change of the metastatic burden can be calculated, even though the exact value of the total number of metastases is unknown. Thus, a more detailed approach would not bring additional insights unless more parameters are available. The parameters for the different simulations were chosen to cover a broad range of possible behaviors of different tumors.

The model was simulated in a simple Python v3 program that is published in a Zenodo repository [26]. This is publicly available to enable simulations of different scenarios. The system of ordinary differential equations is an inhomogeneous one. The inhomogeneities are, on the one hand, that the release of single and clustered CTCs can vary over time, and on the other hand, that the device can be switched on and off or the efficiency can also vary over time. For both aspects, functions (subroutines) are implemented that allow in the present form constant and constant plus sinusoidal circadian changes of release and on–off behavior with freely selectable time windows for the device, respectively. If a more sophisticated model for either the release or device behavior is needed, the program can be adapted easily by exchanging the corresponding function.

The simulations presented here clearly show two main problems for the application of extracorporeal devices:

1. The dynamics of CTC release and death is rather high;
2. The fraction of blood that can be cleared per unit time is rather low.

This is even more severe due to the fact that clusters have an even higher dynamic and a higher metastatic potential [24]. As clearly visible in Figure 2, if the device is switched on temporarily, the reduction in single and clustered CTCs is clearly visible but the concentration recovers within some hours due to the high dynamics, rendering the reduction in the metastatic burden negligible. Even if the device is used overnight during the maximum of the release, or even if the device operates continuously, the blood cannot be 100% cleared, as the fraction of blood in a peripheral vein that can be used is limited. Thus, most of the blood passes through the body via other routes, for example, through the radial vein. It has to be mentioned that there are some additional effects that were not taken into account that are even more unfavorable for an extracorporeal device. Different organs have different preferred localizations of distant metastases. As a rule, a distinction is made between the portal vein type and the cava type. The portal vein type includes malignant tumors of the organs of the digestive tract (lower esophagus, stomach, pancreas, small and large intestine, and upper rectum), which preferentially metastasize to the liver because their venous blood first reaches the liver via the portal vein and then the inferior vena cava. Alternatively, lymphogenous metastases and metastases from the peritoneum may occur. Most other types of cancer belong to the cava type. They are most likely to metastasize to the skeleton, brain, and lungs, but also to the liver and spleen. Thus, especially for the big clusters, it is more likely to get stuck and either die or extravasate during the first passage of the next capillary bed. Thus, an extracorporeal device will miss all the large clusters that get stuck during the first turnover of blood through the vascular system, which might be the majority of clusters.

**Figure A9:**
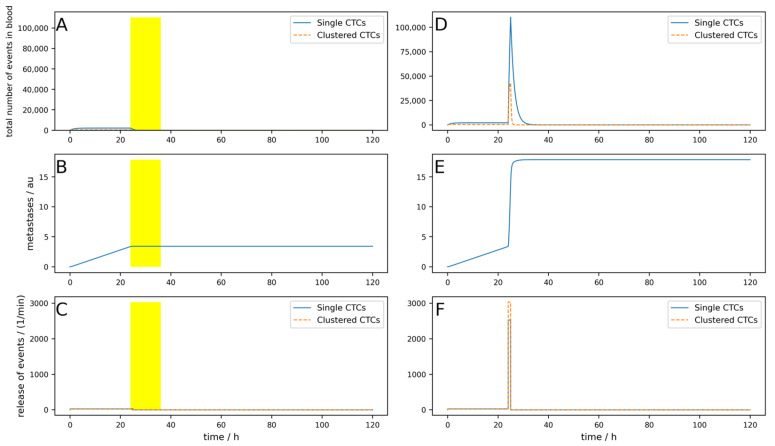
Simulation of an increased release of CTCs due to a tumor removal surgery over 5 days (surgery takes place after 24 h). (**A**–**C**) An implantable device that clears venous blood at the tumor-draining vessel for 12 h after surgery during increased CTC release (indicated by the yellow areas). (**D**–**F**) Control without clearing device using the same parameters. (**A**,**D**) Behavior of single and clustered CTCs in absolute numbers. (**B**,**E**) Metastatic burden in arbitrary units. (**C**,**F**) Release of single and clustered CTCs from the primary tumor.

**Figure A10:**
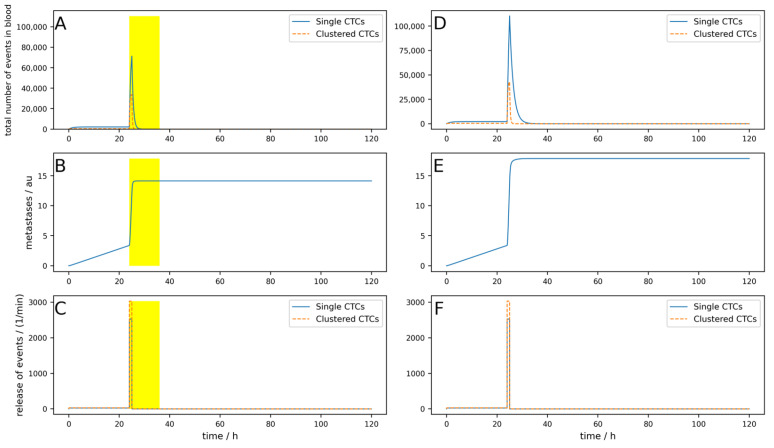
Simulation of an increased release of CTCs due to a tumor removal surgery over 5 days (surgery takes place after 24 h). (**A**–**C**) An extracorporeal device that clears peripheral blood for 12 h after surgery during increased CTC release (indicated by the yellow areas). (**D**–**F**) Control without clearing device using the same parameters. (**A**,**D**) Behavior of single and clustered CTCs in absolute numbers. (**B**,**E**) Metastatic burden in arbitrary units. (**C**,**F**) Release of single and clustered CTCs from the primary tumor.

**Figure A11:**
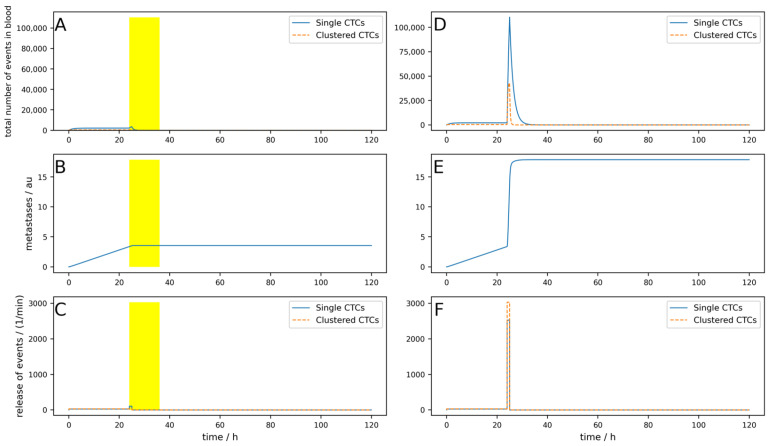
Simulation of an increased release of CTCs due to a tumor removal surgery over 5 days (surgery takes place after 24 h). (**A**–**C**) An implantable device with low efficiency that clears venous blood at the tumor-draining vessel for 12 h after surgery during increased CTC release (indicated by the yellow areas). (**D**–**F**) Control without clearing device using the same parameters. (**A**,**D**) Behavior of single and clustered CTCs in absolute numbers. (**B**,**E**) Metastatic burden in arbitrary units. (**C**,**F**) Release of single and clustered CTCs from the primary tumor.

If, however, the device can be implanted right at the tumor-draining vessel, as proposed in some works [22,23], the effect is rather different. Due to the position, the device virtually reduces the effective release into the blood compartment and then further acts on the circulating CTC events. This, and the fact that such a device should work virtually permanently, immediately increases the clearance to over 98%. Even on a long-term scale where metastases start to produce CTC events themselves, the implantable device still works well. Of course, as mentioned above, the rate of metastasis formation is unknown, and thus also the total number of secondary tumors. The qualitative behavior can also clearly be seen. We assumed a rather high rate and an onset of the release after 20 days. In the absence of a device or if the device is extracorporeal, one can see the progressive growth of metastases and the increasing release of CTC events in Figure 6. Of course, these cells can stay dormant for years, which means that the delay would be much longer than the 20 days used here. However, this would only delay the progressive growth of the metastases and has no direct impact on the efficiency of the device. It would therefore depend on how long the device is used and how long it can operate at high efficiency. Investigating a long dormancy period would be an interesting point for future research. The implantable device significantly delays the onset of progressive growth and could therefore be of therapeutic interest. Certainly, it can now be argued that the cost and difficulty of such an implantation would exceed the cost of a simple surgical removal of the tumor. However, it may not be possible to remove a tumor easily and safely. Moreover, in some cases, surgery to remove the tumor leads to an increased number of CTCs, and thus to a higher risk of metastasis [34]. In such specific cases, a CTC-clearing device would certainly be advantageous. For example, such a device could be implanted in the tumor-draining vessel within the same surgery to eliminate the increased number of CTCs after surgery. Such a case was simulated in Figure A9, Figure A10 and Figure A11 in Appendix A. Figure A9 simulates an implanted device which is turned on for 12 h after tumor removal surgery. The surgery takes place at 24 h, and for one hour after surgery, the CTC release is 100 times higher than normal. All other parameters are the same as used in the simulation shown in Figure A1. It can be seen that the short-term drastic increase in the metastatic burden is almost completely compensated by the device compared to the control without the device. In Figure A10, an identical situation was simulated with an extracorporeal device; here, the reduction in metastatic burden was significantly lower than with the implantable device, which was to be expected. In addition, Figure A11 shows an identical situation again, but this time with a low-efficiency implantable device (80% single and 90% clustered CTCs). The reduction in metastatic burden is slightly worse than with the good implantable device, but still better than with the extracorporeal device, which was also to be expected.

Nevertheless, a different picture emerges if one considers an additional possible pathway for the CTCs, for example, via the lymphatic system. This leads to the lymphatic system acting as a bypass around the device. The greater the proportion of CTCs that are transported via the lymphatic system, the poorer the performance of the device. This leads to an implantable device acting more and more like an extracorporeal device, as it no longer has access to all the newly shed CTCs. It is therefore very important to know how high the proportion of CTCs via the lymphatic system, for example, really is. Unfortunately, there are currently little or no data from the literature on this, but it would be of great importance for these considerations.

Furthermore, the parameter *M* allows metastases to produce CTCs. As recent tumor and metastasis phylogeny studies have clearly demonstrated that metastases may derive from both the primary tumor (primary seeding) as well as established metastatic lesions (secondary seeding) [35], this parameter allows for experimentation with different rates. The results of our simulations have also shown (Figure 7C,D) that this parameter can be important for a CTC-clearing device, as it can act as a bypass for an implantable device similar to the lymphatic system.

**Figure A12:**
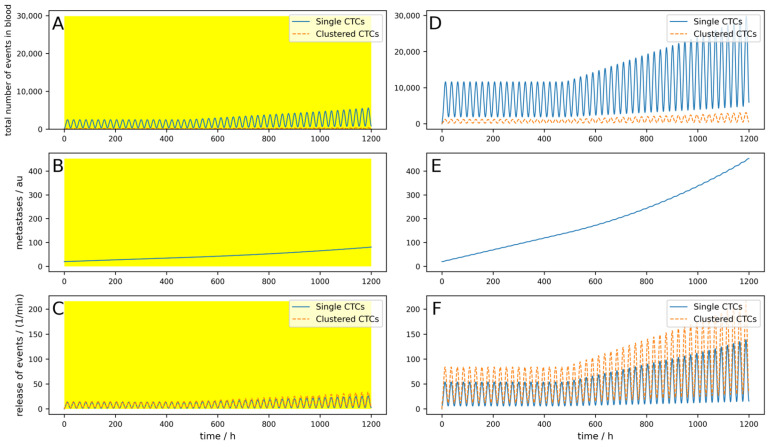
A 50-day simulation of an implantable device where the metastases can emit CTCs and an initial metastatic burden of 20 au. (**A**–**C**) A device that clears venous blood at the tumor-draining vessel is simulated. The device works 24 h a day (indicated by the yellow area). (**D**–**F**) Control without clearing device using the same parameters. (**A**,**D**) Behavior of single and clustered CTCs in absolute numbers. (**B**,**E**) Metastatic burden in arbitrary units. (**C**,**F**) Release of single and clustered CTCs from the primary tumor and the metastases.

Of course, such a device would be most useful in patients in whom cancer dissemination has not yet occurred. But the model can theoretically also deal with the situation where metastases are already present, which certainly can be the case. For this purpose, an initial condition for the metastasis load *m*(*t*) not equal to zero can be set. In combination with parameter *M* being greater than zero, these pre-existing metastases would again act as a bypass and therefore lower the efficiency of reducing the metastatic burden. Figure A12 in Appendix A simulates this case with an initial metastatic burden of 20 au and *M* = 0.01; the other parameters are identical as the ones used for the simulation in Figure 5. It can be seen that in this case, the reduction in metastatic burden of an implantable device reduces to only 82% compared to the 99% reduction in Figure 5.

It should be noted that only devices with a high efficiency above 0.9 were simulated. If the device used for CTC elimination has a low efficiency (i.e., the device does not work well and is therefore unable to remove the vast majority of single and clustered CTCs), the reduction in metastases decreases significantly. It can therefore be concluded that the device for removing CTCs from the blood must have a high efficiency in order to considerably reduce the metastatic burden. The model now makes it possible to simulate different devices with arbitrary efficiencies using the two parameters *eff_dev_* and *eff_devc_* and to predict their performance with regard to metastasis formation.

## 5. Conclusions

The main parameters that determine if a blood-clearing device can reduce metastases are as follows:

- Is the device implanted or extracorporeal?
- The dynamics of single and clustered CTCs;
- The ratio of blood flow through the device and total heart minute volume;
- To what extent are CTCs transported via the lymphatic system, for example, thus potentially bypassing the device?

Other parameters hardly affected the reduction in metastases. For example, whether the device is able to remove 90% or 100% of the CTCs during a passage has scarcely any effect on the formation of metastases.

The basic idea of this work is to provide a simple, extensible model to bridge the gap between clinical science and theoretical modeling. Even if some parameters are not currently known or are difficult to estimate, the model can provide an indication of which parameters are of crucial importance and which are not. It is intended to provide an initial starting point and support for future research in the field of CTCs and metastasis in all aspects, helping to identify relevant parameters and simulate their effects on this complex system. We hope that the simple model described here, including the open access Python v3 code provided, will help to quickly evaluate ideas and concepts of medical devices that aim for the reduction in metastatic burden due to single and clustered CTCs.

## Figures and Tables

**Table 1:** Parameters for the simulations shown in Figure 2, Figure 3, Figure 4, Figure 5 and Figure 6 and Figure A1, Figure A2, Figure A3, Figure A4, Figure A5, Figure A6, Figure A7 and Figure A8. Black letters indicate simulations of extracorporeal devices clearing peripheral blood; blue letters show simulations of implanted devices. A yellow background indicates a long-term simulation with metastases that emit CTCs with time delay Δ*t* = 20 days and *M* = 0.01. All other parameters not listed were not changed within the presented simulations. The parameters for the lymphatic system were set to zero up to now.

Sim.	*r_const_* in min^−1^	*r_dyn_* in min^−1^	*p_const_* in min^−1^	*p_dyn_* in min^−1^	*k_dead_*	*k_deadc_*	*N_c_*	*N_cdev_*	*T_dev_* in h	Δ*t_dev_* in h	*R_vol_*	*eff_dev_*	*eff_devc_*	Red. of Met. in %
Figure 2	25	0	30	0	0.0046	0.023	0	0	3	0	0.10	0.9	0.99	12.6
Figure A1	25	0	30	0	0.0115	0.069	0	0	1	0	0.02	0.9	0.99	1.2
Figure A2	25	0	30	0	0.0046	0.023	0	0	3	0	0.10	1.0	1.00	12.7
Figure 3	5	40	10	60	0.0046	0.023	0	0	3	0	0.10	0.9	0.99	21.1
Figure A3	5	40	10	60	0.0115	0.069	0	0	3	0	0.10	0.9	0.99	14.1
Figure A4	5	40	10	60	0.0115	0.069	0	0	3	8	0.10	0.9	0.99	5.5
Figure A5	5	40	10	60	0.0046	0.023	0	0	8	0	0.10	0.9	0.99	47
Figure A6	5	40	10	60	0.0046	0.023	1	15	8	0	0.10	0.9	0.99	44.6
Figure A7	5	40	10	60	0.0046	0.023	0	0	24	0	0.10	0.9	0.99	81.9
Figure 4	5	40	10	60	0.0046	0.023	0	0	24	0	0.10	0.9	0.99	99.8
Figure A8	5	40	10	60	0.0115	0.069	1	15	23	0	0.02	0.9	0.99	98.1
Figure 5	5	40	10	60	0.0115	0.069	1	15	24	0	0.02	0.9	0.99	99
Figure 6	5	40	10	60	0.0115	0.069	1	15	8	0	0.02	0.9	0.99	11

## Data Availability

The original data presented in the study are openly available in the Zenodo repository Python code for the paper: “Simulating the effect of removing circulating tumor cells (CTCs) from blood reveals that only implantable devices can significantly reduce metastatic burden of patients” at https://doi.org/10.5281/zenodo.13302215 (accessed on 8 July 2024).

## Appendix A

**Table A1:** Parameters for the simulations shown in Figure A1, Figure A2, Figure A3, Figure A4, Figure A5, Figure A6, Figure A7, Figure A8, Figure A9, Figure A10, Figure A11 and Figure A12 in the Appendix A. Black letters indicate simulations of extracorporeal devices clearing peripheral blood; blue letters show simulations of implanted devices. A yellow background indicates long-term simulation with metastases that emit CTCs with time delay Δ*t* = 20 days, *M* = 0.01 and an initial metastatic burden *m*(*t* = 0) = 20. All other parameters not listed were not changed within the presented simulations. The parameters for the lymphatic system were set to zero for the simulations in Appendix A.

Sim.	*r_const_* in min^−1^	*r_dyn_* in min^−1^	*p_const_* in min^−1^	*p_dyn_* in min^−1^	*k_dead_*	*k_deadc_*	*N_c_*	*N_cdev_*	*T_dev_* in h	Δ*t_dev_* in h	*R_vol_*	*eff_dev_*	*eff_devc_*	Red. of Met. in %
Figure A1	25	0	30	0	0.0115	0.069	0	0	1	0	0.02	0.9	0.99	1.2
Figure A2	25	0	30	0	0.0046	0.023	0	0	3	0	0.10	1.0	1.00	12.7
Figure A3	5	40	10	60	0.0115	0.069	0	0	3	0	0.10	0.9	0.99	14.1
Figure A4	5	40	10	60	0.0115	0.069	0	0	3	8	0.10	0.9	0.99	5.5
Figure A5	5	40	10	60	0.0046	0.023	0	0	8	0	0.10	0.9	0.99	47
Figure A6	5	40	10	60	0.0046	0.023	1	15	8	0	0.10	0.9	0.99	44.6
Figure A7	5	40	10	60	0.0046	0.023	0	0	24	0	0.10	0.9	0.99	81.9
Figure A8	5	40	10	60	0.0115	0.069	1	15	23	0	0.02	0.9	0.99	98.1
Figure A9	25 (2500)	0	30 (3000)	0	0.0115	0.069	0	0	1	0	0.02	0.9	0.99	80.9
Figure A10	25 (2500)	0	30 (3000)	0	0.0115	0.069	0	0	1	0	0.02	0.9	0.99	20.9
Figure A11	25 (2500)	0	30 (3000)	0	0.0115	0.069	0	0	1	0	0.02	0.8	0.90	80.1
Figure A12	5	40	10	60	0.0115	0.069	1	15	24	0	0.02	0.9	0.99	82.1
